# Targeting Kupffer Cell Enolase 1 Attenuates Liver Inflammation and Injury in Hemorrhagic Shock

**DOI:** 10.3390/ijms26178340

**Published:** 2025-08-28

**Authors:** Zhijian Hu, Jingsong Li, Naureen Rashid, Asha Jacob, Ping Wang

**Affiliations:** 1 Center for Immunology and Inflammation, The Feinstein Institutes for Medical Research, Manhasset, NY 11030, USA; zhu1@northwell.edu (Z.H.); jli42@northwell.edu (J.L.); nrashid@northwell.edu (N.R.); 2 Departments of Surgery and Molecular Medicine, Zucker School of Medicine at Hofstra/Northwell, Manhasset, NY 11030, USA

**Keywords:** enolase 1, hemorrhagic shock, cleaved caspase 1, inflammation, glycolysis, ENOblock

## Abstract

Hemorrhagic shock (HS) is a type of hypovolemic shock and is a leading cause of mortality worldwide. Enolase 1 (ENO1), a key enzyme in glycolysis, has been implicated in the pathogenesis of inflammatory disorders. We hypothesize that Kupffer cell (KC) ENO1 contributes to liver inflammation and that inhibiting ENO1 with ENOblock protects the liver from HS-induced injury. HS was induced in mice by lowering mean arterial pressure to 25 mmHg for 90 min, followed by fluid resuscitation. Twenty-four hours later, KCs were isolated. To mimic HS in vitro, KCs were isolated from healthy mice and exposed to hypoxia/reoxygenation (H/R). Hypoxic KCs were treated with ENOblock during reoxygenation, and cytokines (IL-1β, TNF-α, IL-6) were measured. In mice subjected to HS and treated with ENOblock, the liver was harvested. In KCs isolated from HS mice as well as in H/R exposed KCs, ENO1 mRNA and protein expression were significantly increased. In KCs exposed to H/R as well as in liver tissues from HS mice, cytokine mRNA and protein levels (IL-1β, TNF-α, IL-6) were increased; however, ENOblock treatment significantly decreased these parameters. HS also markedly increased ENO1 activity and cleaved caspase-1 in KCs, while these parameters were significantly attenuated by ENOblock treatment. These findings suggest that targeting ENO1 in KCs could be a promising therapeutic strategy for mitigating HS-induced liver injury.

## 1. Introduction

Hemorrhagic shock (HS) is a critical and life-threatening condition and remains one of the leading causes of mortality in emergency medicine. In the United States alone, it is responsible for approximately 60,000 deaths annually and contributes to 30–40% of trauma-related fatalities worldwide [1]. The severity of hemorrhagic shock is closely associated with the systemic inflammatory response. Previous research has established that when blood loss exceeds 30–40% (classified as Class III hemorrhage or higher), a pronounced inflammatory cascade is triggered, potentially leading to progressive organ dysfunction and, if left untreated, to fatal outcomes [2,3]. Among the affected organs, the liver is particularly vulnerable due to its high metabolic activity and extensive vascularization. Although its lobular structure and glycogen reserves provide some degree of compensation for hypovolemia, prolonged hypoxia, oxidative stress, and the inflammatory surge upon resuscitation significantly compromise hepatic endothelial cell integrity and hepatocyte viability. These factors render the liver highly susceptible to HS-induced injury, further exacerbating systemic inflammation and organ failure [4,5].

Enolase is a multifunctional moonlighting protein with diverse roles, including gene regulation, mitochondrial membrane stabilization, and facilitation of cancer metastasis [6,7,8]. Its most well-established function is as a key glycolytic enzyme, catalyzing the conversion of 2-phosphoglycerate into phosphoenolpyruvate, a crucial step in glycolysis [9]. Among the enolase family members, enolase 1 (*ENO1*) is ubiquitously expressed across various tissues, whereas enolase 2 (*ENO2*) is primarily found in the brain, and enolase 3 (*ENO3*) is predominantly expressed in muscle tissue [10]. Due to its essential role in glycolysis, *ENO1* has been proposed as a potential therapeutic target in cancer research [6,11]. Moreover, *ENO1* is frequently reported to be upregulated under hypoxic conditions in both clinical and experimental settings [12,13,14]. Beyond its metabolic function, *ENO1* has been implicated in various inflammatory diseases, including endothelial cell inflammatory injury, ulcerative colitis-like inflammation, pulmonary inflammation, and fibrosis [15,16,17,18]. Its involvement in inflammation highlights its potential as a therapeutic target for HS-induced injuries.

Inhibition of ENO1 has been shown to confer beneficial effects in inflammatory diseases, with anti-ENO1 strategies being widely reported [16,17]. Unlike antibody-based approaches, ENOblock is a recently developed small-molecule inhibitor designed to investigate the moonlighting functions of enolase [19,20]. It has been demonstrated to reduce enolase enzyme activity in injured liver tissue in obese mice [21]. The introduction of ENOblock has been associated with an increased population of anti-inflammatory M2 macrophages in adipose tissue [8]. Additionally, it has been shown to attenuate the expression of inflammatory cytokines, prevent hepatocyte steatosis, mitigate liver fibrosis, and reduce hepatocyte apoptosis [8,21]. Although *ENO1* is recognized as a pro-inflammatory mediator upregulated during hypoxic events, its role in HS-induced injury has not been elucidated. Therefore, we investigated the expression and activity of ENO1 in HS, focusing on the liver as a target organ and utilizing ENOblock as an inhibitor. Given that sex hormones can influence immune and metabolic responses, the study was conducted only in male mice. The findings demonstrated that HS led to the upregulation and activation of *ENO1* in KCs, which contributed to inflammasome-dependent KC pyroptosis, leading to liver injury by promoting pro-inflammatory responses. Notably, inhibition of ENO1 protein with ENOblock effectively attenuates post-HS liver inflammation and injury and KC pyroptosis, highlighting its potential as a therapeutic target.

## 2. Results

### 2.1. ENO1 Was Increased in KCs from HS Mice

**Figure 1:**
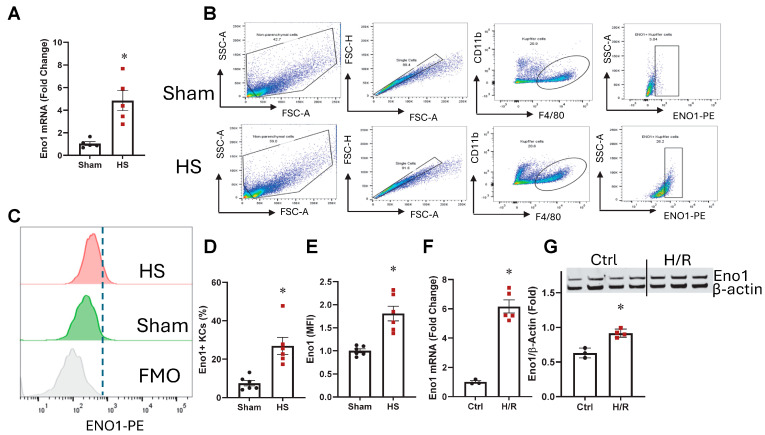
ENO1 was increased in KCs after HS. KCs were isolated from Sham and HS mice, and *ENO1* mRNA (**A**) was assessed by qPCR. Isolated KCs were stained with the surface marker F4/80 and CD11b, along with intracellular ENO1, and ENO1 protein expression (**B**–**E**) was analyzed by flow cytometry. Results are displayed as mean ± SEM (*n* = 5–6/group). KCs were isolated from healthy mice and subjected to H/R, and *ENO1* mRNA (**F**) was measured by qPCR, while protein expression (**G**) was assessed by Western blot. Results are displayed as mean ± SEM (*n* = 3–5/group). All data were subjected to statistical analysis by an unpaired *t*-test. * *p* < 0.05 vs. Sham.

KCs were isolated, and both mRNA and protein expression were assessed. KCs from both sham and HS mice at 24 h after resuscitation were seeded in six-well plates, washed with PBS 1 h after seeding, and then collected for quantification of ENO1 mRNA expression levels. Compared to the sham group, ENO1 mRNA levels increased by 4.8-fold in HS KCs (Figure 1A). Using flow cytometry, KCs were identified as cells expressing F4/80^hi^ and CD11b^lo^ [22]. ENO1 protein level was determined by detecting its PE-conjugated fluorescence signal within the F4/80^hi^ and CD11b^lo^ KCs (Figure 1B,C). Compared to the sham group, the frequency and the mean fluorescence intensity (MFI) of ENO 1 protein in KCs were significantly elevated by 3.6-fold and 1.8-fold following HS, respectively (Figure 1D,E).

### 2.2. ENO1 Was Upregulated in Kupffer Cells After H/R

KCs were isolated, exposed to hypoxia (1% O_2_ and 5% CO_2_ at 37 °C) overnight, followed by 2 h of reoxygenation. Afterward, the cells were harvested to assess ENO1 expression at both mRNA and protein levels. Following H/R, ENO1 mRNA and protein expression levels increased by 6.2-fold and 1.5-fold, respectively (Figure 1F,G).

### 2.3. ENOblock Alleviates H/R-Induced Inflammatory Injury

**Figure 2:**
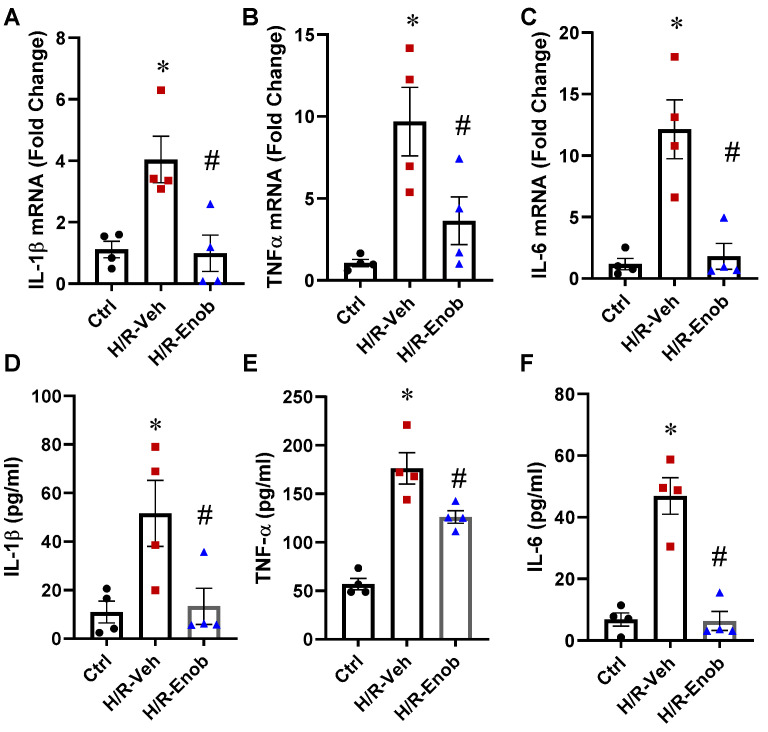
ENOblock alleviates H/R-induced Kupffer cell inflammation. KCs were subjected to H/R, after which the cells and the supernatant were harvested. Total RNA from the cells was extracted to assess the mRNA expression of IL-1β (**A**), TNF-α (**B**), and IL-6 (**C**) by qPCR. The supernatant collected was used for cytokine analysis. The levels of IL-1β (**D**), TNF-α (**E**), and IL-6 (**F**) were measured using an ELISA kit. Results are displayed as mean ± SEM (*n* = 4–6/group) and were subjected to statistical analysis through ANOVA and the SNK test. * *p* < 0.05 vs. Sham; # *p* < 0.05 vs. HS-Veh.

In KCs, following overnight exposure to hypoxia (1% O_2_) and 2 h of reoxygenation, with or without 5 µM ENOblock treatment, cells and supernatants from each group were collected to assess pro-inflammatory cytokine expression levels. After H/R, IL-1β, TNF-α, and IL-6 mRNA levels increased by 3.6-fold, 9.1-fold, and 10.3-fold, respectively, compared to the control group. Notably, ENOblock treatment attenuated these increases by 76%, 63%, and 85%, respectively (Figure 2A–C). The supernatant levels of IL-1β, TNF-α, and IL-6 were also significantly elevated by 4.7-fold, 3.1-fold, and 6.9-fold, respectively (Figure 2D–F). However, ENOblock treatment effectively reduced these cytokines by 74%, 28%, and 86%, respectively.

### 2.4. ENOblock Alleviates HS-Induced Liver Inflammation and ENO1 Activity

**Figure 3:**
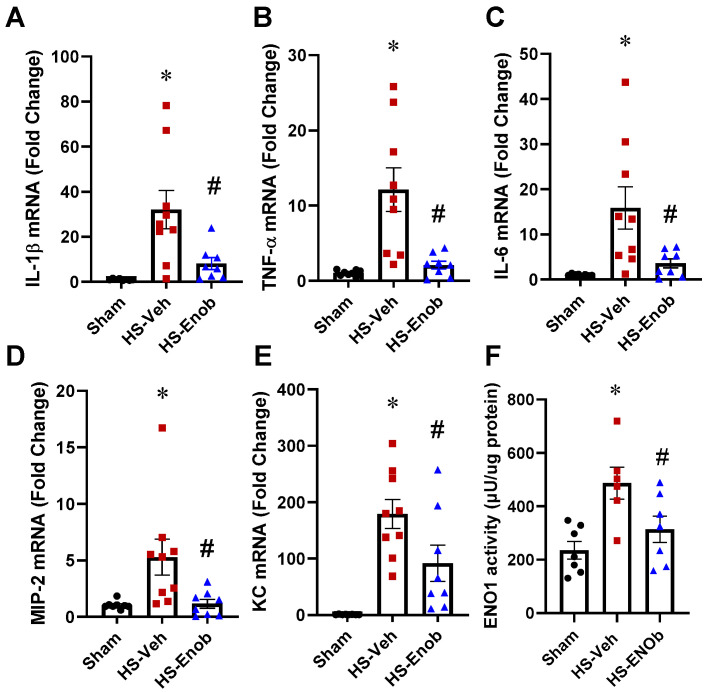
ENOblock alleviates liver inflammation and ENO1 activity in HS. Liver tissues from Sham and HS mice treated with either Vehicle or ENOblock were harvested, and mRNA was extracted for analysis. The mRNA expression of cytokines IL-1β (**A**), TNF-α (**B**), and IL-6 (**C**), as well as chemokines MIP-2 (**D**) and KC (**E**), was measured by qPCR. Additionally, KCs were isolated from different groups, and ENO1 glycolytic activity was determined by an enolase activity assay kit (**F**). Results are presented as mean ± SEM (*n* = 8–9/group for mRNA assessment, *n* = 6–7/group for activity assay) and were subjected to statistical analysis through ANOVA and the SNK test. * *p* < 0.05 vs. Sham; # *p* < 0.05 vs. HS-Veh.

To further assess the impact of ENOblock on HS-induced tissue injury, liver samples from each group were analyzed. The mRNA expression of IL-1β, TNF-α, and IL-6 was increased by 30.6-fold, 11.3-fold, and 15.6-fold (Figure 3A–C), respectively, in HS samples compared to the sham group. However, ENOblock treatment significantly mitigated these increases by 78%, 83%, and 77%, respectively. Further evaluation of pro-inflammatory chemokines revealed that MIP-2 and KC mRNA expression levels were elevated by 5.1-fold and 171-fold following HS (Figure 3D,E). Notably, ENOblock administration reduced their expression by 78% and 49%, respectively. Additionally, the enzymatic activity of ENO1 in Kupffer cells from HS and sham animals was evaluated, demonstrating a 2.2-fold increase in the HS group compared to the sham group (Figure 3F). However, treatment with ENOblock resulted in a 32% reduction in ENO1 activity.

### 2.5. ENOblock Mitigates Liver Injury in HS

**Figure 4:**
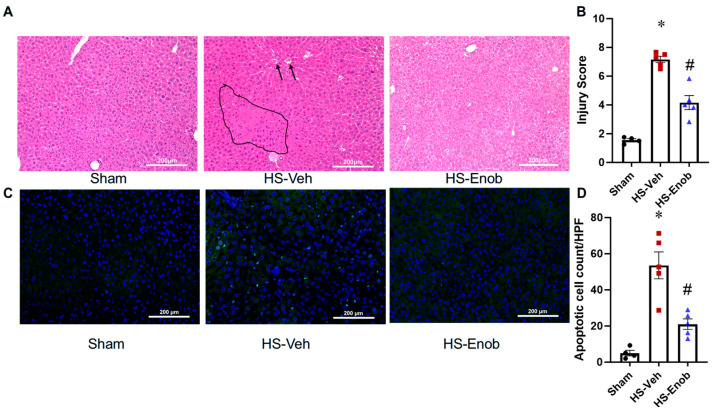
ENOblock mitigates liver injury in HS. Liver tissue samples from different groups were subjected to H&E staining (**A**), and representative liver necrosis (circled area) and congestion (arrow pointed) are marked. Injury scores (**B**) were evaluated from H&E staining based on the Suzuki criteria. TUNEL staining (**C**) was performed to assess apoptosis, and the quantification of positive stained cells (**D**) was conducted using ImageJ software. All stained images were captured at ×200 magnification. Results are displayed as mean ± SEM (*n* = 4–5/group) and were subjected to statistical analysis through ANOVA and the SNK test. * *p* < 0.05 vs. Sham; # *p* < 0.05 vs. HS-Veh.

Histological analysis of H&E-stained liver sections was conducted to evaluate tissue injury. Images from randomly selected fields were systematically scored. In liver samples from HS animals, histopathological alterations included sinusoidal congestion, hemorrhage, extensive cellular swelling, increased eosinophilia, cytoplasmic vacuolation, faint or absent nuclei, and focal areas of hepatic necrosis. However, these pathological changes were markedly attenuated in HS mice treated with ENOblock (Figure 4A). Based on the average Suzuki injury scores, the scores increased by 4.6-fold following HS, whereas ENOblock treatment significantly reduced the score by 42% (Figure 4B). These histological findings correlated with TUNEL assay results (Figure 4C), which quantified apoptotic cells in each section. HS induced a 10.7-fold increase in TUNEL-positive stained cells, whereas ENOblock treatment significantly reduced apoptotic signals by 61% (Figure 4D).

### 2.6. ENOblock Attenuates C-caspase-1 in KCs After HS

**Figure 5:**
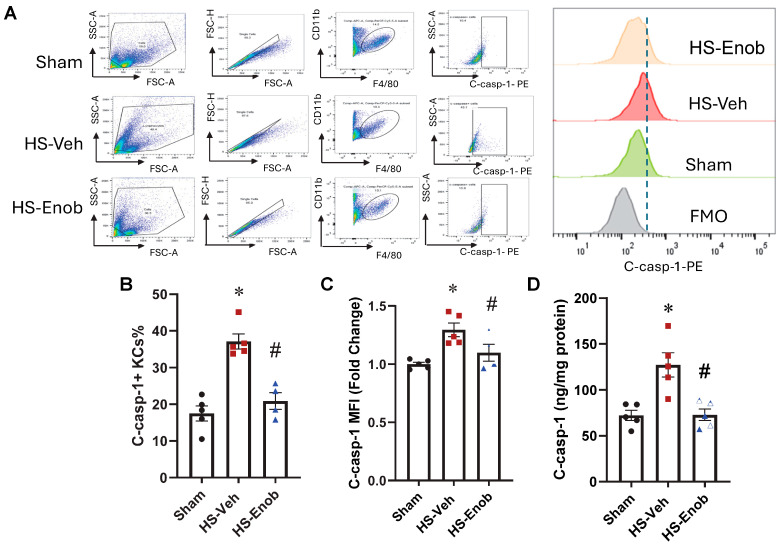
ENOblock attenuates C-caspase-1 in KCs after HS. KCs were isolated and stained with surface marker F4/80 and CD11b, along with intracellular c-Caspase-1, and expression of c-Caspase-1 (**A**) was analyzed by flow cytometry. The data are shown as the percentage of c-Caspase-1-positive cells (**B**) and fold change in c-Caspase-1 mean fluorescence intensity (MFI) (**C**). KCs were isolated to determine C-caspase-1 levels using an ELISA kit (**D**). Results are displayed as mean ± SEM (*n* = 4–5/group) and were subjected to statistical analysis through ANOVA and the SNK test. * *p* < 0.05 vs. Sham; # *p* < 0.05 vs. HS-Veh.

To further investigate the mechanism underlying ENO1-mediated inflammatory injury in the liver, the expression level of C-caspase-1 was assessed in KCs isolated from the sham and HS mice with or without ENOblock treatment. KCs were isolated and stained with fluorophore-conjugated antibodies to evaluate c-Caspase-1 expression using flow cytometry (Figure 5A). Compared to the sham group, the frequency and the MFI of c-caspase-1 positive cells were significantly elevated by 2.1-fold and 1.3-fold, respectively, following HS. However, ENOblock treatment reduced these levels by 44% and 15% (Figure 5B,C). This finding was further validated by measuring c-Caspase-1 levels in KCs from sham and HS mice with or without ENOblock treatment, using an ELISA kit. c-Caspase-1 levels were increased by 1.8-fold compared to the sham group; however, ENOblock treatment reduced this elevation by 43% (Figure 5D).

## 3. Discussion

Despite the restoration of hemostasis and correction of hypoperfusion, secondary organ injury and dysfunction often arise due to the systemic inflammatory response triggered by global hypoxia and subsequent resuscitation during HS. The liver, being highly susceptible to post-HS injury, remains a critical target; however, the underlying mechanisms are not yet fully understood. As a key enzyme in the glycolysis pathway, ENO1 expression is upregulated under hypoxic conditions [6]. It has been shown that enhanced glycolysis drives immune cell activation and promotes pro-inflammatory responses [23]. Analysis of KCs from liver tissue in the HS mice revealed a marked increase in ENO1 expression at both the mRNA and protein levels. These findings were further confirmed in Kupffer cells subjected to H/R. To elucidate the role of ENO1 in Kupffer cell inflammatory responses, we utilized ENOblock, a specific inhibitor of ENO1, which has been demonstrated to suppress its glycolytic activity [8,24,25]. The data demonstrated that H/R-induced activation of Kupffer cells led to a substantial upregulation of pro-inflammatory cytokines IL-1β, TNF-α, and IL-6. ENOblock treatment significantly decreased these cytokines, which is consistent with previous findings that ENO1 activation induces the release of IL-1β, TNF-α, and IL-6 [10]. In addition, gene expressions of these cytokines, along with chemokines MIP-2 and KC, were also increased in the liver tissues of HS mice, and ENOblock treatment significantly decreased these parameters, suggesting the role of ENO1 in inflammatory responses in the liver after HS. These findings aligned with those of other studies, which reported that ENOblock treatment downregulated hepatic ENO1 activity [21].

As inflammation persists, hepatic injuries ensue, as evidenced by progressive hepatic damage, including hepatocyte swelling, vacuolation, karyolysis, and patchy necrosis observed through histological examination of liver tissue, which is often seen in HS [4,26]. Additionally, TUNEL staining demonstrated a significant increase in apoptotic cells, further highlighting the extent of liver damage in HS mice. The inhibition with ENOblock markedly attenuated liver tissue damage as shown by histological analysis, and reduced the number of TUNEL-positive apoptotic cells. This is consistent with previous findings that ENOblock reduces inflammatory cytokine expression, alleviates liver injury, and decreases steatosis, apoptosis, and fibrosis [8,21]. Therefore, the increases in ENO1 expression and activity, alongside inflammation and tissue injury, strongly indicate its involvement in HS-induced inflammation and hepatic injury.

The precise mechanism by which ENOblock exerts protection in the liver after HS has not been completely elucidated. ENO is an enzyme that catalyzes the conversion of 2-phosphoglycerate to phosphoenolpyruvate in the glycolysis pathway. It is ubiquitously expressed, and α-enolase or ENO1 is differentially expressed in human pathologies, implicating ENO1 as an indicator of tissue dysfunction in multiple diseases [27]. ENO1 has diverse secondary moonlighting functions that are unrelated to its catalytic activity, such as binding plasminogen on the cell membrane, stabilizing the mitochondrial membrane, and acting as a repressor of gene expression in the nucleus [28]. ENOblock was first developed to elucidate the moonlighting functions of ENO1 in biological assays [20,28]. ENOblock inhibited the pathology of diet-induced obesity by acting as a transcriptional repressor of master regulators of lipid homeostasis (Srebp-1a and Srebp-1c), gluconeogenesis (Pck-1), and inflammation (TNF-α and IL-6). This study suggests that ENOblock binds ENO1 at the dimerization domain and induces nuclear localization, where it acts as a transcriptional repressor [8,21]. In our study, ENOblock significantly decreased H/R- and HS-induced pro-inflammatory responses and HS-induced liver inflammation and injury.

Activated immune cells such as macrophages undergo metabolic reprogramming, which is characterized by a reduction in oxidative phosphorylation alongside an increase in glycolysis, influencing their function and phenotype [29,30]. Glycolysis is essential for pro-inflammatory cells like M1 macrophages, while oxidative metabolism promotes the development of anti-inflammatory M2 macrophages [31]. Blocking pyruvate kinase M2, a crucial enzyme in glycolysis, inhibits metabolic programming in macrophages by decreasing M1 polarization [32]. It has been demonstrated that M1-polarized macrophage aggregation, accompanied by NLRP3 inflammasome activation and an increase in cleaved Caspase-1 (c-Caspase-1), GSDMD, and IL-1β, plays a crucial role in HS-induced injury [33]. The release of active IL-1β further amplifies inflammation and induces pyroptosis [34]. It has also been reported that enhanced glycolysis triggers NLRP3 inflammasome activation, whereas glycolysis inhibition downregulates pyroptosis-related genes [35,36,37]. Given that HS activates NLRP3 inflammasomes, which in turn mediate pyroptosis [38], we investigated whether blocking ENO1 with ENOblock attenuates KC pyroptosis. HS led to a significant increase in c-Caspase-1, a key mediator in NLRP3 inflammasome-mediated pyroptosis, in KCs isolated from HS mice, which was attenuated by ENOblock treatment. Notably, effective inhibition of ENO1 expression and activity with ENOblock significantly mitigated liver injury and improved prognosis, possibly by decreasing KC pyroptosis.

During HS, the liver, which has a rich blood supply, is highly susceptible to ischemia and hypoxia, leading to liver dysfunction. Although rapid hemostasis and fluid resuscitation are crucial steps in the recovery process, these steps are significant contributors to the occurrence of reperfusion injury. Therefore, in the ischemic stage, hepatocytes are damaged by hypoxia due to the cessation of blood flow; meanwhile, in the reperfusion phase, those hepatocytes that were already damaged are further damaged. The massive production of oxygen free radicals, calcium overload, Kupffer cell activation, inflammatory response, and mitochondrial dysfunction, which occur during the reperfusion stage, exacerbate hepatocellular injury [39,40,41]. Since HS is an inflammatory response indication, immune cells such as the macrophages should play a crucial role in its mechanistic manifestation. Kupffer cells, which are resident macrophages in the liver, are mostly responsible for the observed inflammatory response in HS. Thus, Kupffer cells play a crucial role in damaging the hepatocytes during HS. Therapies developed against excessive activation of Kupffer cells could attenuate the inflammatory response and subsequent liver injury. The current findings indicate that Kupffer cell ENO1 is at least in part responsible for the exaggerated inflammatory response observed in HS and that the ENO1 inhibitor ENOblock was effective in reducing liver inflammation and injury. Therefore, ENOblock could be developed in the future as a therapeutic for hemorrhage-induced liver injury.

While developing therapeutics for hemorrhagic shock, the interrelationship between hepatocytes and KCs, as well as other cells in the liver, should be considered. Studies on the interrelationships among cell populations have been extremely challenging due to the difficulty in purifying different cell populations in the liver. However, single-cell transcriptomic technologies have enhanced our understanding of acute liver injury and liver regeneration by providing detailed insights into cellular heterogeneity, dynamic changes, and cell–cell cross talk [42]. These approaches have uncovered novel molecular mechanisms in key cell populations such as hepatocytes, macrophages, endothelial cells, and hepatic stellate cells, thereby contributing to understanding the mechanism of hepatic injury and repair. In addition, transcriptomic data may not fully capture the complexity of the mechanisms. The development of single-cell omics, such as proteomics and metabolomics, is needed to achieve a comprehensive understanding of the mechanism underlying liver injury during HS, as well as other forms of liver injury and repair processes.

These findings provide compelling evidence that ENO1 mediates HS-induced inflammation and hepatic injury at least in part by inflammasome-dependent KC pyroptosis. However, several limitations should be acknowledged. First, since the study relied solely on pharmacological inhibition using ENOblock, genetic validation is essential to exclude off-target effects of ENOblock. In this regard, a study reported that knocking down ENO1 in dendritic cells (DCs) using lentiviral siRNA technology had exhibited impaired maturation and activation of DCs with a significant decrease in intracellular pyruvate concentration compared to wild-type DCs. This study suggested that enhanced glycolysis is required for efficient antigen processing and presentation of DCs to induce a robust immune response [43]. Similar knockdown approaches in KCs can be conducted in the future to confirm our findings of the pharmacological inhibition of ENO1 by ENOblock. Therefore, the lack of studies in KC-specific knockout models is one of the limitations of our study. Second, our experiments were conducted exclusively in male mice. Given that sex hormones can influence immune and metabolic responses, it is important to include both males and females in this study. Future dose and time response studies and additional pharmacokinetic studies will be conducted in both sexes. Third, the observed findings regarding the role of ENO1 are primarily based on observations of KC responses in the liver under conditions of HS and H/R. Since HS is a systemic ischemic injury affecting multiple organs, HS induction may activate other immune cells that significantly contribute to the systemic inflammatory response, and therefore, it is important to assess the effect of ENOblock in additional organs. Enoblock has never been studied in HS. Since the liver is the most vital organ affected by HS and the current study is a preliminary approach to examine the effect of Enoblock in HS, the liver was the chosen organ for this study. Thus, to elucidate the systemic relevance of our findings, in future studies, in addition to the liver, both the lungs and the kidneys will be included. Fourth, to develop ENOblock as a therapeutic for hemorrhagic shock, pharmacokinetics in preclinical and safety studies in humans are certainly needed in the future. Fifth, it can be argued that the study did not fully dissect which specific function of ENO1 is responsible for the observed detrimental effects. In this regard, a study showed that ENOblock suppressed KC glycolysis and proliferation [24]. In addition, another study showed that ENOblock reduces enolase activity in vivo, induces nuclear translocation of enolase, and produces anti-diabetic, anti-inflammatory, and anti-fibrotic effects [21]. Therefore, while we have not explored the actual mechanism of action of ENO1 in HS, ENO1 could work via multiple fronts in HS. Sixth, since the current study is a preliminary approach to examining the effect of ENOblock in HS, only a short-term evaluation was conducted. Therefore, our observation is limited to 24 h post-resuscitation, and long-term assessments are needed to understand ENOblock’s sustained effects. Future studies will include extended time points as well as survival studies. Additionally, species-specific variations in cellular responses and molecular mechanisms must be considered. While C57BL/6 mice serve as a widely accepted preclinical model, there may be potential discrepancies when extrapolating these findings to clinical settings.

## 4. Materials and Methods

### 4.1. Enolase1 Inhibitor

Enolase 1 inhibitor AP-III-a4 (ENOblock, C_31_H_44_ClFN_8_O_3_) was obtained from Selleck Chemicals LLC (Houston, TX, USA) in lyophilized form (Cat. No. S7443). For experimental use, ENOblock was reconstituted in cell culture-grade water to the desired concentration.

### 4.2. Experimental Animals

Male C57BL/6 mice were obtained from the Jackson Laboratory (Bar Harbor, ME, USA) at 7 weeks of age. Upon arrival, the mice were housed under controlled conditions with a 12 h light/dark cycle and were provided unrestricted access to standard Purina mouse chow and water. The animals were acclimated for a minimum of 7 days prior to use in experiments. Experimental procedures were conducted on mice between 8 and 12 weeks of age. All animal handling and experimental protocols were approved by the Institutional Animal Care and Use Committee (IACUC) of The Feinstein Institute for Medical Research and were carried out in strict compliance with the Guide for the Care and Use of Laboratory Animals.

### 4.3. Mouse Model of Hemorrhagic Shock

Mice were randomly assigned to sham, vehicle, and treatment groups. Hemorrhagic shock was induced using previously established protocols [44,45]. Mice were anesthetized via intraperitoneal administration of pentobarbital sodium (50 mg/kg) and positioned supine on a surgical plane. Bilateral femoral arteries were carefully dissected and cannulated with heparinized PE-10 polyethylene tubing (BD, Sparks, MD, USA). The cannula in the left femoral artery was connected to a blood pressure analyzer (BPA; Digi-Med, Louisville, KY, USA) for continuous monitoring of blood pressure, while the cannula in the right femoral artery was used for blood withdrawal, shock pressure regulation, administration of treatment compounds, and fluid resuscitation. Shock was induced by gradually withdrawing blood through the right femoral artery until the mean arterial pressure (MAP) reached 25 ± 3 mmHg, which was maintained for 90 min to establish the shock condition. Following the shock phase, mice in the treatment group were resuscitated with Ringer’s lactate solution containing ENOblock at a dosage of 5 mg/kg, infused at a rate of 2 mL/h. Mice in the vehicle group received an equivalent volume of Ringer’s lactate solution supplemented with saline. The total volume of the resuscitation fluid was twice the volume of shed blood. After resuscitation, the cannulas were removed, and surgical incisions were appropriately closed. Mice were monitored postoperatively and returned to their cages upon full recovery from anesthesia. At 24 h following resuscitation, mice were re-anesthetized, blood was collected, and upon euthanasia, the liver was harvested for subsequent analyses.

### 4.4. Kupffer Cell Isolation

Kupffer cells from both HS mice and healthy mice were isolated using a modified type IV collagenase digestion method based on previously reported protocols [46]. Following anesthesia with isoflurane inhalation, the inferior vena cava was cannulated with PE-50 tubing as the afferent channel, while an incision was made in the portal vein to serve as the efferent outlet. The liver was first perfused with 10 mL of pre-warmed Hank’s Balanced Salt Solution (HBSS) containing 0.5 mM EGTA through the PE-50 tubing, followed by perfusion with pre-warmed HBSS (Cat. No. 14175095; Thermo Fisher Scientific, Waltham, MA, USA) supplemented with 1 mM CaCl_2_ and 0.5 mg/mL type IV collagenase (Cat.: LS004188; Worthington Biochemical, Lakewood, NJ, USA) at a rate of 120 mL/h.

After perfusion, the liver was excised and transferred to a 100 mm cell culture dish, where it was minced into small pieces in RPMI 1640 medium (Cat. No. 11875; Thermo Fisher Scientific) containing 0.25 mg/mL type IV collagenase. The tissue was further homogenized into a cell suspension using a 10 mL syringe and incubated at 37 °C for 30 min, with homogenization performed every 15 min. Following complete digestion, the liver homogenate was filtered through a 70 μm cell strainer. The filtrate was centrifuged at 300× *g* for 5 min at 4 °C, and the supernatant was discarded. The pellet was resuspended in 10 mL of RPMI 1640 medium, and centrifugation was repeated under the same conditions. The isolated cells were then resuspended in 10 mL of RPMI 1640 medium and subjected to centrifugation at 50× *g* for 3 min at 4 °C. The supernatant was collected and further centrifuged at 300× *g* for 5 min at 4 °C to obtain the non-parenchymal cells. The final cell suspension was prepared in complete RPMI 1640 medium supplemented with 10% fetal bovine serum (FBS), 2 mM glutamine, and 1% penicillin–streptomycin. For flow cytometry analysis, the cell suspension was washed with fluorescence-activated cell sorting (FACS) buffer and processed accordingly. For culturing, the cell suspension was seeded onto tissue culture plates, followed by washing with phosphate-buffered saline (PBS) 1 h after seeding to remove contaminating non-adherent cells. Since the non-adherent cells were washed off quickly from the plate within 1 h after seeding, it is most likely that the adherent cells remaining in the plate were Kupffer cells. In fact, it is possible that the entire Kupffer cell population may not have been recovered using this procedure. No additional KC markers were used in the isolation procedure to minimize any mechanical stress on the cells prior to exposing them to overnight hypoxia.

### 4.5. Hypoxia/Reoxygenation In Vitro

Isolated KCs were cultured overnight in complete RPMI 1640 medium, washed in PBS, resuspended in Opti-MEM medium, and exposed to hypoxia (1% O_2_ and 5% CO_2_ at 37 °C) overnight, followed by 2 h of reoxygenation. In some experiments, during the 2 h reoxygenation, the cells were treated with either PBS (Vehicle) or 5 µM ENOblock. After H/R and treatment, the cells and supernatant were collected for further analysis.

### 4.6. Enolase Activity Assay

Enolase activity was assessed using the Enolase Assay Kit (ab241024, Abcam, Toronto, ON, Canada) following the manufacturer’s instructions. Prior to the assay, cells were washed thoroughly with ice-cold PBS and subsequently lysed in ice-cold assay buffer. The cell suspension was then harvested and centrifuged at 12,000 rpm for 5 min at 4 °C, and the supernatant was collected for enzymatic analysis. Enolase activity was measured at 25 °C using a fluorometric kinetic assay (Ex/Em = 535/587 nm) with a reading interval of 4 min. The activity levels were calculated as milliunits/mL using a standard curve ranging from 0–1000 pmol H_2_O_2_ and were normalized with protein concentration.

### 4.7. Flow Cytometry

Following harvesting, KCs were washed with fluorescence-activated cell sorting (FACS) buffer and stained with PerCP/Cy5.5 anti-mouse CD11b antibody (Cat. No. 101228, Biolegend, San Diego, CA, USA) and APC anti-mouse F4/80 antibody (Cat. No. 123116, BioLegend). The cells were then fixed using Fluorofix buffer (Cat. No. 422101; BioLegend, San Diego, CA, USA), followed by intracellular staining for Enolase 1 (Cat. No. MA5-32756; Thermo Fisher Scientific) or cleaved caspase-1 (Cat. No. 4199s; Cell Signaling Technology, Danvers, MA, USA). Intracellular staining was performed through incubation with the respective primary antibodies, followed by staining with a PE-conjugated secondary antibody in permeabilization wash buffer (Cat. No. 406421; BioLegend).

### 4.8. Western Blot

Frozen liver tissue was pulverized into a fine powder and lysed in RIPA buffer supplemented with protease and phosphatase inhibitors. Protein extraction was performed by centrifugation at 12,000× *g*, and protein concentrations were determined using the Bradford protein assay reagent (Bio-Rad, Hercules, CA, USA). Equal amounts of protein samples were resolved via polyacrylamide gel electrophoresis (PAGE) and subsequently transferred onto nitrocellulose membranes (Invitrogen, Waltham, MA, USA; Thermo Fisher Scientific). Following membrane transfer, blocking was performed using 0.1% casein in TBS. The membranes were then incubated with primary antibodies against Enolase 1 (Cat. No. MA5-32756; Thermo Fisher Scientific). Antibody incubation solutions were prepared in TBS containing 0.1% casein and 0.1% Tween-20. Subsequently, the membranes were washed and incubated with the corresponding secondary antibodies. Labeled protein bands were visualized using the Odyssey CLx Imaging System (Li-Cor Biosciences, Lincoln, NE, USA) and analyzed with ImageJ software (https://imagej.net/software/imagej/).

### 4.9. Cytokines and C-caspase-1 Measurement

The concentrations of interleukin-6 (IL-6) (Cat. No. 555240; BD Biosciences) in serum and cell culture supernatant, as well as tumor necrosis factor (TNF-α) (Cat. No. 558534; BD biosciences) and interleukin-1β (IL-1β) (Cat. No. DY401-05; Biotechne, Minneapolis, MN, USA) levels in the supernatant, were determined using enzyme-linked immunosorbent assay (ELISA) kits (BD Biosciences, San Jose, CA, USA) following the manufacturer’s protocols. The expression of cleaved caspase-1 (c-Caspase-1) in isolated KCs after HS was determined using ELISA kits (Cat. No. AG-46B-0003-KI01; Adipogen Life Sciences, San Diego, CA, USA) following the manufacturer’s protocols.

### 4.10. Real-Time Quantitative PCR

Total RNA was extracted from liver tissue using TRIzol reagent (Invitrogen, Waltham, MA, USA), followed by phase separation with BCP (1-Bromo-3-chloropropane) and precipitation with isopropanol. For cell samples, RNA extraction was performed using a commercial RNA isolation kit according to the manufacturer’s instructions. The purified RNA was then reverse transcribed into complementary DNA (cDNA) using murine leukemia virus reverse transcriptase (Applied Biosystems, Waltham, MA, USA). Subsequently, quantitative polymerase chain reaction (qPCR) was conducted using Power SYBR Green PCR Master Mix (Applied Biosystems) with gene-specific forward and reverse primers. Amplification and real-time fluorescence detection were performed using the StepOnePlus Real-Time PCR System (Applied Biosystems, Waltham, MA, USA). mRNA expression levels were quantified using the 2^−ΔΔCt^ method, with β-actin mRNA serving as an internal reference. Results are expressed as fold changes relative to the sham group. The primer sequences used for qPCR are as follows:

Enolase1, forward 5′-CTGGCCAAGTACAATCAGATCCTC-3′ and reverse 5′-GGATCTCCGGTCCATGCTTTA-3′
interleukin (IL)-6, forward 5′-CCGGAGAGGAGACTTCACAG-3′ and reverse 5′-CAGAATTGCCATTGCACAAC-3′;
IL-1β, forward 5′-CAGGATGAGGACATGAGCACC-3′, and reverse 5′-CTCGCAGACTCAAACTCCAC-3′
TNF-α, forward 5′-AGACCCTCACACTCAGATCATCTTC-3′ and reverse 5′-TTGCTACGACGTGGGCTACA-3′;
KC, forward 5′-GCTGGGATTCACCTCAAGAA-3′ and reverse 5′-ACAGGTGCCATCAGAGCAGT-3′;
macrophage inflammatory protein (MIP)-2, forward 5′-CATCCAGAGCTTGAGTGTGA-3′ and reverse 5′-CTTTGGTTCTTCCGTTGAGG-3′;
β-actin, forward 5′-CGTGAAAAGATGACCCAGATCA-3′ and reverse 5′-TGGTACGACCAGAGGCATACAG-3′.

### 4.11. Liver Injury Histopathological Evaluation

After harvesting, liver tissues were fixed in 10% formalin, followed by paraffin embedding. The embedded specimens were then sectioned into 5 μm-thick slices and mounted onto glass slides. Hematoxylin and eosin (H&E) staining was performed on the sections to facilitate histopathological evaluation. For each sample, four randomly selected fields were captured under light microscopy for injury assessment. Liver injury scores were determined based on the Suzuki criteria as previously described by us [47], which evaluate three histological parameters: congestion (none, 0; minimal, 1; mild, 2; moderate, 3; severe, 4), vacuolization (none, 0; minimal, 1; mild, 2; moderate, 3; severe, 4), and necrosis (none, 0; single-cell necrosis, 1; ≤30%, 2; 30–60%, 3; >60%, 4). Each image was independently scored, and the scores were subsequently averaged to obtain the final liver injury score for each sample.

### 4.12. Terminal Deoxynucleotidyl Transferase dUTP Nick End Labeling (TUNEL) Assay

Paraffin-embedded liver tissue sections were deparaffinized, rehydrated, and permeabilized using proteinase K. The sections were then stained with a freshly prepared TUNEL reaction mixture from the In Situ Cell Detection Kit (Roche Diagnostics, Indianapolis, IN, USA) and incubated at 37 °C for 30 min. TUNEL signals were visualized and captured using a fluorescence microscope (EVOS FL Auto Imaging System, Thermo Fisher Scientific). Signals were recorded from three randomly selected fields per sample. The apoptotic TUNEL signals were subsequently quantified using ImageJ software.

### 4.13. Statistical Analysis

Data presented in the figures are expressed as the mean ± standard error of the mean (SEM). For multiple-group comparisons, one-way analysis of variance (ANOVA) was performed, while an unpaired *t*-test was used for two-group analyses. Statistical significance was defined as *p* < 0.05. For post hoc multiple comparisons, the Student–Newman–Keuls (SNK) test was conducted to assess inter-group differences. All statistical analyses and graphical representations were performed using GraphPad Prism software Version 8 for Windows (www.graphpad.com).

## 5. Conclusions

In conclusion, we selected the liver as the target organ to investigate potential mechanisms underlying HS-induced organ injury and to explore effective strategies for reducing HS-related inflammation and injury. Our study identified ENO1, a key glycolytic enzyme, as being overexpressed in response to HS-induced hypoperfusion and resuscitation, leading to a significant increase in glycolysis. This metabolic shift was found to activate the inflammasome-dependent pathway mediators such as c-Caspase-1, ultimately resulting in KC pyroptosis. Importantly, inhibiting ENO1-mediated glycolysis with ENOblock effectively attenuated KC pyroptosis, highlighting the pivotal role of ENO1 in HS-induced liver inflammation and injury. Given the fact that targeting inflammasome activation through glycolysis modulation has been demonstrated as an effective strategy in the treatment of various inflammatory diseases [36], regulating glycolytic imbalance presents a promising therapeutic approach for improving HS outcomes.

## Data Availability

Data will be made available upon reasonable request.

## Abbreviations

The following abbreviations are used in this manuscript:

HS	Hemorrhagic shock
ENO	Enolase
KCs	Kupffer cells
H/R	Hypoxia/reoxygenation
mRNA	Messenger RNA
TUNEL	Terminal deoxynucleotidyl transferase dUTP nick end labeling
IL-1β	Interleukin-1beta
TNF-α	Tumor necrosis factor-alpha
IL-6	Interleukin-6
MAP	Mean arterial pressure
HBSS	Hank’s balanced salt solution
FBS	Fetal bovine serum
FACS	Fluorescence-activated cell sorting
PBS	Phosphate-buffered saline
PAGE	Polyacrylamide gel electrophoresis
TBS	Tris-buffered saline
ELISA	Enzyme-linked immunosorbent assay
BCP	1-bromo-3-chloropropane
qPCR	Quantitative polymerase chain reaction
KC	Keratinocyte-derived cytokine
MIP-2	Macrophage inflammatory protein-2
H&E	Hematoxylin and eosin
SEM	Standard error of the mean
ANOVA	Analysis of variance
SNK	Student–Newman–Keuls
